# #Propage l'info, pas le virus : communication politique et réponses des influenceur.euses à l'appel du gouvernement Legault lors de la crise de la COVID-19 au Québec

**DOI:** 10.1017/S0008423921000536

**Published:** 2021-05-25

**Authors:** Frédérique Côté, Mireille Lalancette

**Affiliations:** 1 Étudiante au deuxième cycle–Communication sociale, Département de lettres et communication sociale, Université du Québec à Trois-Rivières, 3351 boulevard des Forges, Trois-Rivières, QC G8Z 4M3; 2 Professeure en communication politique et membre du Groupe de recherche en communication politique (GRCP), Département de lettres et communication sociale, Université du Québec à Trois-Rivières, 3351 boulevard des Forges, Trois-Rivières, QC G8Z 4M3

**Keywords:** COVID-19, communication gouvernementale, médias socionumériques, célébrité, influenceur, COVID-19, government communication, digital media, celebrity, influencer

## Abstract

En mars 2020, le premier ministre Legault a fait appel aux influenceur.euses et aux célébrités québécoises dans le cadre de la campagne #Propage l'info, pas le virus afin de sensibiliser les jeunes au respect des consignes sanitaires liées à la COVID-19. Cet article offre un éclairage inédit sur les différentes manières dont ces personnes renommées ont répondu à l'appel ainsi que sur les formes de leurs réponses à l'aide d'une analyse de contenu de leur vidéo partagée sur les réseaux sociaux. Le codage des vidéos s'est fait à partir d'une grille d'analyse qualitative de contenu, inspirée de celle de Fields (1988). Il ressort des analyses que différents moyens ont permis d'accentuer le sentiment de proximité entre la célébrité et son public, dans le but d'augmenter l'adhésion au message. L'utilisation du pronom « On », l'emploi de formules narratives et l'intimité qui se dégage des vidéos informatives vont en ce sens.

## Abstract (EN)

In March 2020, Premier Legault called on Quebec influencers and celebrities as part of the #Propage l'info, pas le virus campaign to educate youth about following health guidelines against COVID-19. This article provides an insight into the different ways in which these renowned individuals responded to this call as well as the forms of their responses. To do so, the content of the videos shared by these individuals on social networks was analyzed using a qualitative coding scheme inspired by Fields (1988). The result of this analysis shows that different means were used to accentuate the feeling of closeness between the celebrity and his audience, in order to increase the adherence to the message. The use of the pronoun “On”, the use of narrative formulas and the intimacy that emerges from informative videos may contribute to the rapport between a celebrity and his public.

La communication politique de crise du gouvernement du Québec a pris plusieurs formes depuis le début de la pandémie liée à la COVID-19. C'est au mois de mars 2020, alors que la propagation du virus est encore relativement faible sur le territoire, que le gouvernement québécois instaure la routine des conférences de presse diffusées en direct à la population grâce aux médias numériques et d'information. La plupart du temps présidées par le premier ministre François Legault, les conférences de presse ont fait intervenir différent.es ministres et responsables en fonction des sujets qui étaient abordés ce jour-là. L'ancienne ministre de la Santé, madame Danielle McCann ainsi que le responsable de la santé publique, docteur Horacio Arruda, ont tenu un rôle important pendant les premiers mois de la crise. Il faut dire que le trio avait, à un certain moment, rendez-vous avec la population québécoise pratiquement chaque jour pour faire le bilan de la situation, en plus de présenter les mesures spéciales et les plans d'action.

Malgré la répétition des mises en garde afin de minimiser la propagation de la COVID-19, le gouvernement Legault a dû faire face à de nombreuses difficultés, par rapport au respect des mesures de confinement et de la distanciation physique. En effet, les adolescent.es du Québec ont mis du temps à accepter et à respecter celles-ci, alors que les mesures devenaient de plus en plus strictes. C'est pourquoi, lors de la conférence de presse du 17 mars 2020, Monsieur Legault a fait appel aux « leaders jeunes » pour qu'eux fassent passer le message, en les interpellant de cette façon :

[…] Je veux lancer un message à tous les leaders jeunes, que ce soit les artistes, les sportifs. Vous allez probablement être meilleurs que moi pour lancer cet appel-là. Ça peut être, entre autres, par les médias sociaux. S'il vous plaît, dites aux jeunes au Québec de respecter les consignes. […] On va lancer une campagne aussi, le gouvernement. La campagne va s'appeler […] « Propage l'info, pas le virus » […] (Legault, 2020).

Dans cet article, nous nous intéressons à la réponse de ces « leaders jeunes » qui sont en fait des influenceurs.euses et célébrités québécoises. Plus précisément, nous nous demandons comment ceux.celles-ci ont répondu à l'appel du premier ministre. Cet article offre ainsi un éclairage inédit sur les différentes manières dont ces personnes renommées ont répondu à l'appel ainsi que sur les formes de leurs réponses. Cette réflexion est pertinente alors que peu de recherches ont été réalisées en lien avec le rôle des célébrités en communication politique au Canada (Jackson & Darrow, 2005; Lalancette & Cormack, 2020; Lalancette & Raynauld, 2019) et encore moins au Québec. Ce faisant, cette recherche permet un regard novateur sur les nouvelles formes de campagnes politiques et sur leurs potentiels impacts chez des publics précis. L'appui des célébrités dans le présent cas est d'autant plus riche à étudier compte tenu du contexte extraordinaire dans lequel il se retrouve, soit en temps de crise.

## Célébrités

Au cœur de cette étude, il y a la notion de célébrité. On dit qu'une personne est une célébrité lorsqu’« elle est très connue et qu'elle entretient une relation émotionnelle avec le public » [traduction libre] (Marland & Lalancette, 2014, 133). Autrement dit, ces personnes font l’« objet d'une [forte] attention collective » (Hourmant et al., 2019a, 12), ou de ce qui est appelé par d'autres une accumulation de « capital d'attention » (Rojek, 2003). Cette attention est intensifiée par la médiatisation (Strömbäck & Van Alest, 2013). Celle-ci est une composante principale de la célébritisation selon Driessens (2012). Dans l’« ère de la communication mondiale sans limites où la culture du divertissement se fond sans encombre avec la haute culture, le pouvoir des célébrités semble plus puissant, plus connecté et davantage en harmonie et en phase que le pouvoir électoral » [traduction libre] avancent même Hart et Tindall (2009, 256). Ils ajoutent à cet effet que « l'un et l'autre [sont] alignés et se renforcent ainsi mutuellement ».

Dans le cadre de cet article et par rapport à la définition précédemment rapportée, on entend par célébrité les influenceur.euses populaires grâce aux médias socionumériques, les artistes des milieux culturels – musique, cinéma, télévision, arts – ou de la scène sportive. Par ailleurs, précisons qu'il existe plusieurs types de célébrités. La typologie la plus utilisée est celle de Chris Rojek (2003) qui les divise en trois formes. La première est la célébrité *assignée* qui réfère aux individus qui héritent de leur célébrité comme la royauté, ou bien les enfants de personnes célèbres (pensons par exemple à Justin Trudeau, aux enfants de Johnny Hallyday ou de Céline Dion et René Angelil). La célébrité *acquise*, quant à elle, regroupe les gens qui grâce à leur talent et leurs réalisations – sportives, culturelles, du monde des affaires ou de la politique, par exemple – développent un impact social et possèdent une reconnaissance importante (pensons ici aux gens d'affaires comme le fondateur du Cirque du Soleil Guy Laliberté ou aux sportif.ve.s de haut niveau, comme le nageur Michael Phelps). La troisième forme de célébrité est dite *attribué*, c'est-à-dire lorsqu'elle provient d'un buzz médiatique, par exemple comme lorsqu'une vidéo devient virale. Cette dernière est octroyée par les médias traditionnels ou socionumériques pour des raisons autres que le talent ou les réalisations accomplies. Les participants et participantes des émissions de télé-réalité ou les personnes qui voient leur renommée grandir à la suite de la publication d'une vidéo YouTube, d'un tweet ou d'une publication Facebook ou Instagram qui devient viral en sont de bons exemples.

Retenons ici le rôle central de la communication médiatique dans le processus de célébrification. Comme le résume Barry (2008, 252), « la célébrité est un phénomène culturel à part entière – l'industrie contemporaine est alimentée par la complicité lubrique de la télévision réalité, de l’édition de magazines et des ragots sur Internet. Cela reflète plutôt l'incursion de la célébrité dans tous les domaines de la vie publique : la science, le sport, les arts, la politique et la monarchie » [traduction libre]. Ainsi, tel que le résument Lalancette et Raynauld (2019, 69) :

La capacité d'attraction et d'influence des célébrités n'est ainsi plus à remettre en question. Elles comptent maintenant des millions d'abonnés sur leur compte Twitter, Facebook, Instagram ou SnapChat. Leurs différents statuts sont commentés, aimés (« likés »), redistribués et, dans certains cas, ont un impact direct sur la couverture journalistique des médias traditionnels (Chadwick & Stromer-Galley, 2016). Il n'est donc pas étonnant de constater que plusieurs entreprises s'affairent, par différents moyens, à s'adjoindre aux vedettes sportives, musicales, cinématographiques ou culinaires afin d'améliorer leur image de marque et d'accroître les ventes de leurs produits et services.

De ce fait, il est possible d'affirmer que la célébrité de nos jours tend à devenir de plus en plus numérique (Hourmant et al., 2019a) et les médias socionumériques sont les principaux vecteurs de ce changement. Une personnalité publique n'a plus besoin des médias traditionnels pour se faire connaître et pour « cultiver [sa] visibilité » (Hourmant et al., 2019a, 21), étant donné qu'elle peut le faire elle-même via les nombreuses plateformes socionumériques. Ces pratiques innovantes mènent à la création d'un nouveau type de célébrité appelée « do-it-yourself (DIY) celebrity » (Driessens, 2012, 646). C'est dans cette catégorie que l'on retrouve les influenceur.euses web. On définit l'influenceur.euse comme étant une « personne qui, par son audience sur les réseaux sociaux, est susceptible, par la diffusion de contenu portant sur des sujets divers, d'influencer les comportements de consommation et les opinions des internautes » (OQLF, 2018). Ces célébrités n'ont donc pas nécessairement les mêmes auditoires, puisqu'elles « incarn[ent] à travers leur image publique des groupes sociaux, des communautés ou des valeurs […] » qui rejoignent des segments distincts de la population (Hourmant et al., 2019b, 195). Et c'est peut-être sur ce point particulier que se retrouve toute la richesse de l'endossement d'une célébrité. Ce faisant, une cause, une personne ou un enjeu jouit, par le biais de la célébrité, de l'attention d'un public qui peut être habituellement totalement désintéressé envers ce domaine (Marland & Lalancette, 2014). Plusieurs chercheur.e.s croient qu'une célébrité engagée va générer davantage d'attention sur une cause ou un problème, en plus d'augmenter la visibilité, la crédibilité et le rappel du message (Driessens, 2012; Marland & Lalancette, 2014, notamment).

En fait, la célébrité pourrait aisément assurer le rôle du leader d'opinion, d'après le modèle de Katz et Lazarsfeld (1966) mieux connu sous le nom de la théorie du two-step flow. Cette théorie de la communication de masse est une alternative au modèle classique de stimulus-réponse qui était, jusqu'alors, la seule façon de concevoir l'influence des médias de masse (Willett, 1992). Le modèle classique propose un lien direct entre les médias et le public, composé de centaines, de milliers ou parfois de millions d'individus, et considère que la réception du message provoque chez ces individus une réponse immédiate. De son côté, le modèle en séquence de Katz et Lazarsfeld ajoute un intermédiaire, désigné comme étant le leader d'opinion, entre les médias et le public. Le leader se retrouve au milieu de la chaîne, c'est-à-dire qu'il capte le message diffusé par les médias de masse avant de le transmettre aux membres de son auditoire (voir Graphique 1). C'est d'ailleurs pourquoi on parle d'une communication en deux temps. De plus, « les leaders d'opinion se caractérisent par un plus grand usage des médias, un degré élevé de sociabilité et par le fait qu'ils sont conscients de leur influence sur les autres et de leur rôle de source d'informations et de guide » (Willett, 1992, 328). C'est à travers leurs relations interpersonnelles que les leaders d'opinion sont en mesure de rejoindre leur public. Dans le cas qui nous intéresse, ce sont les médias socionumériques qui sont mobilisés et les abonné.es des plateformes numériques des « leaders jeunes » sont les individus en contact avec les leaders d'opinion qui participent à la campagne #Propage l'info, pas le virus.

**Graphique 1.**
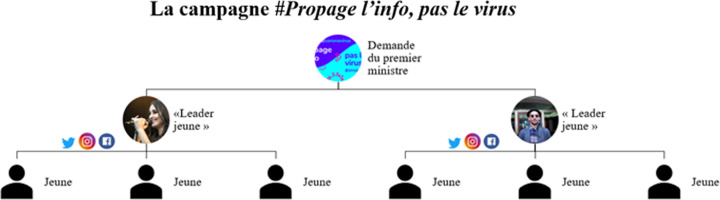
Représentation de la campagne #Propage l'info, pas le virus à partir d'un modèle en séquence

Par cette théorie, il est possible de mieux saisir l'influence qu'a une célébrité lorsque celle-ci se positionne comme leader d'opinion et qu'elle entretient des relations avec son public – sur les réseaux sociaux, par exemple. Elle est la personne désignée pour diffuser un message contenu dans les médias de masse à un public précis, ce qui assure une meilleure réception et réactivité.

## Endossement des célébrités et politique

Dans ce contexte, il n'est donc pas surprenant de constater que les célébrités sont mises à contribution de nombreuses façons et pour différentes raisons, autant dans le marketing commercial (Jackson, 2008) que face à des enjeux sociaux et politiques (Lalancette & Raynauld, 2019). Il est important de souligner que l'appui peut se manifester par la participation à un événement-bénéfice ou par un don monétaire à une cause, comme il peut aussi s'agir d'une prise de position (Driessens et al., 2012). Ainsi, certaines célébrités utilisent leur capital social et culturel afin de prendre part aux débats sociaux, comme celui lié à l'environnement par exemple. Au Québec, en 2018, c'est 400 personnalités publiques qui ont signé le Pacte pour lutter contre les changements climatiques et pour inciter le gouvernement à agir (Shields, 2018). Plus récemment, dans la foulée des événements entourant la mort de George Floyd aux États-Unis, on verra plusieurs célébrités prendre position contre le racisme (Radio-Canada, 29 mai 2020). C'est notamment le cas du jeune chanteur québécois Émile Bilodeau, qui a participé à de nombreuses manifestations pacifiques (Agence QMI, 2020), en plus d'offrir à ses admirateur.trices sur les réseaux sociaux une chanson exclusive – avant qu'elle ne paraisse sur son prochain album – visant à dénoncer le racisme systématique au Québec (compte Instagram *@emile.bilodeau*, 14 octobre 2020).

Tout comme les enjeux sociaux, les partis et leaders politiques sont aussi susceptibles d’être soutenus et défendus publiquement par des célébrités. On se souviendra d'ailleurs de l'appui fortement médiatisé et documenté de la réputée animatrice de télévision Oprah Winfrey au candidat démocrate Barack Obama, lors de la course à la présidence américaine en 2008 (Pease & Brewer, 2008). Au Québec, les élections du gouvernement provincial apportent leur lot d'exemples. C'est notamment le cas en 2012, où un rassemblement du Parti québécois, alors dirigé par Pauline Marois, a rassemblé plusieurs personnes influentes issues de la communauté artistique dont Julie Snyder, Denise Filiatrault et Michel Rivard (White, 2012). On peut aussi penser à la campagne électorale provinciale de 2018, lorsque la jeune actrice Alice Morel-Michaud a passé quelques jours dans l'autobus politique de Québec solidaire en compagnie des candidat.e.s. Elle a documenté le tout en plus d'affirmer ouvertement son vote pour le parti sur les réseaux sociaux (compte Instagram @*alicemorelm*, 28 septembre et 1 octobre 2018). On verra aussi Rosalie Vaillancourt, une humoriste, appuyer fièrement Gabriel Nadeau-Dubois, un candidat de Québec solidaire au moyen d'une publication Instagram (compte Instagram @*rosalievaillancourt*, 25 septembre 2018). Il faut dire que ces dames ne seront pas les seules; plusieurs personnalités publiques afficheront leur couleur cette année-là sur les plateformes socionumériques comme en témoigne la liste de Grondin et Labranche (2018).

Lorsque l'on regarde la littérature à propos de l'endossement politique des célébrités, on constate que les chercheur.es proposent différentes typologies afin de circonscrire et de mieux comprendre le phénomène. Pour Street (2004), il y aurait 1) le politicien ou la politicienne célèbre jouant sur les codes de la célébrité pour rejoindre son électorat et 2) les vedettes décidant de se lancer en politique. Selon Hart et Tindall (2009), on peut distinguer plusieurs types de célébrités : 1) la célébrité activiste, 2) la célébrité qui endosse, 3) la célébrité qui se lance en politique et 4) le.la politicien.ne accédant au statut de célébrité. Quant à Marland et Lalancette (2014, 136), deux types d'endossement sont possibles pour les célébrités. Elles peuvent 1) appuyer des candidat.es, des politicien.nes élu.es ou des partis politiques ou 2) appuyer et faire de la publicité et des collectes de fonds pour ceux.celles-ci. La célébrité qui appuie, donc celle de type 1, regroupe des célébrités qui grâce à leur capital d'attention peuvent obtenir du rayonnement médiatique pour la cause, le.la candidat.e, le.la politicien.ne ou le parti qu'elle soutient. Cette dynamique est profitable dans la mesure où les valeurs sont partagées. La célébrité qui appuie et fait de la publicité (type 2) est souvent une personnalité liée à l’élite médiatique. À cet effet, « [son] engagement prend différentes formes, de la collecte de fonds en passant par le lobby plus direct auprès des décideurs politiques » (Lalancette & Raynauld, 2019, 74).

## Études sur l'endossement en politique

Le sujet suscitant toujours plus d'intérêt au sein de la communauté scientifique, plusieurs chercheur.ses ont tenté de mesurer les effets de ces endossements en étudiant notamment les réactions des personnes de la génération Y, en supposant qu'elles seraient plus susceptibles d’être influencées par les célébrités (par exemple Jackson, 2008; Pease & Brewer, 2008). Nownes (2012) a démontré par le biais d'une expérience regroupant plus de 500 participant.es que les jeunes étaient influencé.es par les activités politiques des célébrités et que celles-ci changeaient leurs perceptions des partis politiques et des causes endossées par celles-ci. Bref, son étude, et celles de collègues tels que Jackson (2008) ainsi que Pease et Brewer (2008), illustrent que lorsque les célébrités s'impliquent en politique, elles peuvent avoir un effet persuasif et que cela peut modifier les attitudes et potentiellement les actions des jeunes. Dans une optique apparentée, Veer et al. (2010) démontrent que les célébrités ont une influence sur les jeunes qui ont déjà une implication politique élevée. Leur étude montre que si les partis politiques misent sur les célébrités afin de cibler les membres du public moins actifs, mais intéressés par la politique, cela peut avoir impact sur leur vote le jour de l’élection. Ici, comme le proposent les théoriciens, le fait de tabler sur le lien plus fort entre les célébrités et le public que celui entre le public et la politique joue un rôle clé du point de vue des processus de persuasion.

Dans la même veine, Becker (2013) a réalisé une étude auprès de jeunes universitaires. Elle y démontre l'impact positif sur les perceptions politiques de l'appui des célébrités à certaines causes et leur endossement politique. Cependant, le lien n'est pas direct. Certains facteurs entrent en jeu comme le démontrent Bergkvist et al. (2016). Ainsi, la perception de la célébrité, les liens entre celle-ci et le sujet promu, son expertise sur le sujet ainsi que la perception des motivations de la célébrité à s'engager ou à endosser une personnalité publique ou une idée jouent un rôle clé dans la persuasion (voir aussi del Mar Garcia de los Salmones et al., 2013). Soulignons encore une fois la place importante des médias socionumériques dans ce processus. Des plateformes comme Instagram, Facebook et Snapchat permettent de communiquer directement avec les fans, et ce, de manière très informelle et interactive. De ce fait, le lien personnel et intime développé par les célébrités avec leurs admirateur.trices peut jouer un rôle sur la manière dont le message est perçu, tel que le démontrent Click et al. (2015).

C'est peut-être en ce sens que pensait également le premier ministre Legault et son équipe de communication lorsqu'ils ont fait appel aux influenceur.euses et aux célébrités québécoises pour les aider à rejoindre les jeunes¹. Ainsi, comment les célébrités ont-elles répondu à l'appel du premier ministre Legault lors de la campagne #Propage l'info, pas le virus? Nous étudierons cet appui accordé à un message du gouvernement à partir d'une étude de cas. Dans ce but, nous allons faire ressortir les manières dont les célébrités québécoises se sont réapproprié le message du gouvernement afin de le transmettre à leur jeune public.

## Étude de cas : la réponse des célébrités à l'appel du gouvernement Legault

Tel qu'expliqué précédemment, le cas qui nous intéresse prend sa source dans la conférence de presse du 17 mars 2020. Durant celle-ci, le premier ministre Legault lança un appel aux artistes et aux influenceur.euses québécois.es afin de diffuser massivement les consignes sanitaires auprès des jeunes via les réseaux socionumériques. La campagne sera d'ailleurs identifiée officiellement à l'aide d'une affiche publicitaire et d'un mot-clic (voir Graphique 2).

**Graphique 2.**
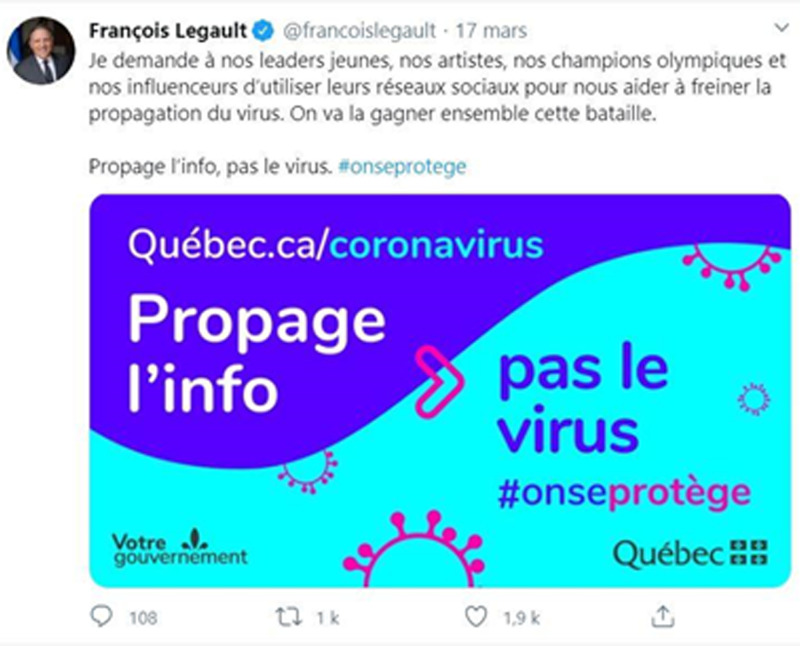
Capture d’écran de la publication qui suit la conférence de presse du 17 mars 2020 sur la page Twitter du premier ministre François Legault

### Corpus

Afin de mieux comprendre la façon dont les personnalités publiques se sont engagées dans la campagne #Propage l'info, pas le virus, nous avons analysé le contenu des publications partagées par ces personnes sur trois différents réseaux socionumériques : Facebook, Instagram et Twitter. Chaque réseau social a ses propres caractéristiques; Facebook mise surtout sur le partage d'informations sous la forme de texte, de photographie ou de vidéo à des ami.es, ce qui forme une communauté. Il est aussi possible de s'abonner à des pages créées pour des célébrités par exemple, ou des entreprises, afin d’être informé des plus récentes nouvelles. De son côté, Instagram est davantage axé sur la publication de contenu photographique et audiovisuel. La dernière plateforme retenue, Twitter, est principalement utilisée par les internautes pour la diffusion d'informations d'intérêt médiatique. Il est autorisé de partager de courts textes, des photographies et des vidéos. De façon générale, les trois plateformes permettent à l'utilisateur.trice de publier du contenu sur son profil, mais aussi de réagir au contenu des personnes auxquelles il.elle est abonné.e en aimant (« like »), commentant ou partageant. On remarquera aussi la présence du premier ministre sur les plateformes retenues, qui a agi à titre d'initiateur de la campagne via ses différents comptes. Cela va sans dire que ces trois réseaux socionumériques sont populaires auprès des jeunes (surtout Instagram) et que les célébrités s'y affichent publiquement (Gruzd & Mai, 2020). Autrement, ces plateformes ont aussi été priorisées pour des raisons méthodologiques étant donné la facilité d'accès aux publications antérieures et la recherche par mot-clic.

La collecte de données sur les réseaux sociaux s'est faite à l'aide des mots-clics suivants : #onseprotège, #onseprotege et #propagelinfopaslevirus. Ceux-ci semblaient les plus pertinents pour l’étude étant donné leur lien direct avec la campagne gouvernementale. Malgré les différences dans les fonctionnalités de recherche des plateformes, les critères de sélection restaient les mêmes. D'abord, il a été décidé de ne retenir que les vidéos en raison de la charge supérieure d'informations contenue dans le multimédia et de la tendance envers ce format de publication. De plus, le compte ayant partagé la vidéo devait avoir 1 000 abonnés et plus. Cette limite inférieure est établie par Jourdain et Doki-Thonon (2019), qui mentionnent qu’« il faut être suivi par un minimum de 1 000 personnes sur une plateforme pour être considéré comme influenceur » (29). Finalement, à partir du contenu de la publication, un lien explicite avec la campagne #Propage l'info, pas le virus devait ressortir. Cela a donc créé un corpus d'analyse de 53 vidéos. La liste des célébrités dont la vidéo a été recensée est présentée à l'Annexe I. Il est à noter que les publications diffusées sur plusieurs plateformes, mais en provenance d'une même personne, n'ont été comptabilisées qu'une seule fois.

## Méthode

Le codage des vidéos s'est fait à partir d'une grille d'analyse qualitative de contenu, inspirée de celle de Fields (1988), initialement créée pour l'analyse qualitative du contenu des nouvelles télévisées. La grille utilisée dans le cadre de notre étude comporte sept axes. D'abord, il est recherché dans la vidéo une mention au gouvernement québécois ou à François Legault. Advenant le cas où ces acteurs sont mentionnés, il faut déterminer le ton, c'est-à-dire si cela est fait de façon positive, négative ou neutre. L’évaluation est faite à partir de l'emploi et de l'utilisation des mots dans l’élocution de la célébrité. Une mention neutre fait simplement référence aux acteurs, comme le démontre l'exemple suivant issu du corpus : « Le gouvernement nous a demandé de passer un message sur les réseaux sociaux […] », via le compte Instagram *@pascal_morisette* (17 mars 2020). D'un autre côté, une mention positive est caractérisée par un commentaire valorisant et élogieux à l’égard du gouvernement ou des personnes qui le représentent (« […] D'ailleurs il fait un superbe boulot, Monsieur Legault. Bravo! Thumbs up! » via le compte Instagram *@neevhumoriste* [17 mars 2020]). La grille rapporte aussi les pronoms de parole dominants afin de mieux comprendre le contact avec le public. Il est à noter que plusieurs pronoms pouvaient être attitrés à une vidéo. Les deux critères qui suivent sont en quelque sorte des stratégies de communication, à savoir si le.la locuteur.trice emploie des formes narratives ou de promotion de soi. Finalement, l'action performée dans la vidéo – une chanson, une mise en scène humoristique, un tour de magie, une prise de parole et bien d'autres – est notée dans le but d'en dégager la visée principale. Les possibilités pour la visée, qui est en fait l'intention derrière la vidéo de sensibilisation, sont le divertissement ou l'information. Pour résumer le tout, voici un aperçu de la grille d'analyse non complétée (voir Graphique 3).

**Graphique 3.**
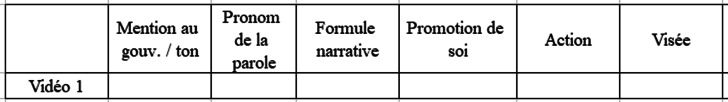
Modèle de la grille d'analyse pour les vidéos

Le codage des vidéos a été effectué par une codeuse unique. Le premier visionnement de chaque vidéo ne servait pas au codage, mais servait plutôt de préparation à celui-ci. Ensuite, il y a eu un visionnement pour chaque composante de la grille puisqu'elles étaient codées individuellement, à l'exception de celles de l'action et de la visée. Comme les deux sont interreliées, ces composantes ont été interprétées en même temps. De plus, au moment d'inscrire les informations pertinentes dans la grille, les vidéos pouvaient être temporairement suspendues puisqu'il n'y a pas eu de transcription intégrale des vidéos. Avant l'analyse des résultats, le codage a été validé par une juge externe à la suite de discussions entre cette dernière et la codeuse afin d'obtenir un consensus.

## Résultats et discussion

La première observation est par rapport à la mention au gouvernement et au ton de celle-ci. Parmi les vidéos recensées, des références au gouvernement québécois ou à son représentant, François Legault, ont été faites à dix-neuf reprises. Parmi les mentions, treize sont faites sur un ton neutre alors que six emploient un ton positif, comme l'illustre le graphique 4.

**Graphique 4.**
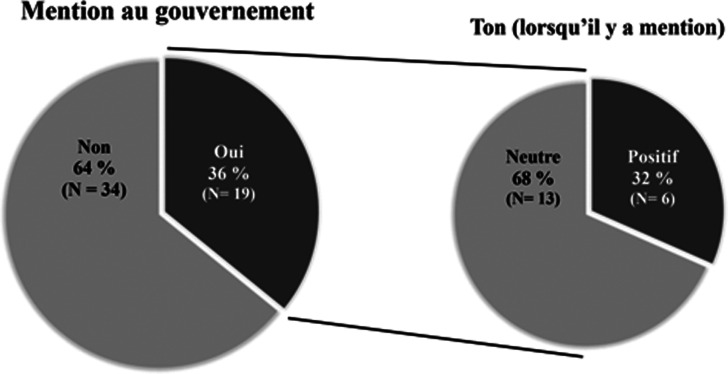
Proportion et fréquence de la mention au gouvernement ainsi que du ton employé lorsqu'il y a mention

En fait, il est possible de dire que l'appui au message politique se manifeste quelque part dans le simple fait de participer à la campagne. Or, en plus de cette preuve, on remarque aussi des commentaires positifs ouvertement faits au gouvernement dans quelques vidéos. En voici un exemple : « Je me trouve choyé d'avoir à la tête des gens si responsables, dégourdis, articulés, lucides dans leurs propos et qui nous guident, je pense, très très bien dans cette mésaventure historique. […] » formulé par l'animateur de l’émission matinale *Salut Bonjour*, Gino Chouinard (compte Facebook *Gino Chouinard*, 17 mars 2020). Ce genre de commentaire peut démontrer un plus fort appui des célébrités envers les acteur.trices politiques en question, que ce soit dans le contexte de la gestion de la crise ou en dehors de celui-ci. De plus, il est important de préciser qu'aucune mention négative n'a été répertoriée. L'intense situation de crise que représente alors l'arrivée du virus de la COVID-19 en sol québécois était peut-être inopportune à la critique. En effet, « formuler des critiques dans ce contexte-là, c'est très mal vu, évidemment. C'est considéré comme un bris de solidarité », explique le sociologue et historien Gérard Bouchard lors d'un entretien avec *Le Devoir* (Bélair-Cirino & Crête, 2020). La solidarité dont il est question ici est la convergence du message communiqué par les différent.es acteur.trices, qu'il.elles soient le gouvernement, l'opposition, les médias ou encore les célébrités. Le mot d'ordre est de rassurer et de protéger la population ainsi, « il serait contre-productif et peut-être irréfléchi, voire criminel d'instiller un doute dans la tête des gens » en critiquant (Professeur anonymisé dans Bélair-Cirino & Crête, 2020).

La prochaine analyse porte sur l'utilisation des pronoms de parole. La fréquence d'usage illustrée par le graphique 5 a révélé que ceux qui reviennent les plus souvent sont les pronoms de la deuxième personne du singulier (*Tu*) et du pluriel (*Vous)*, ainsi que le pronom personnel *On*.

**Graphique 5.**
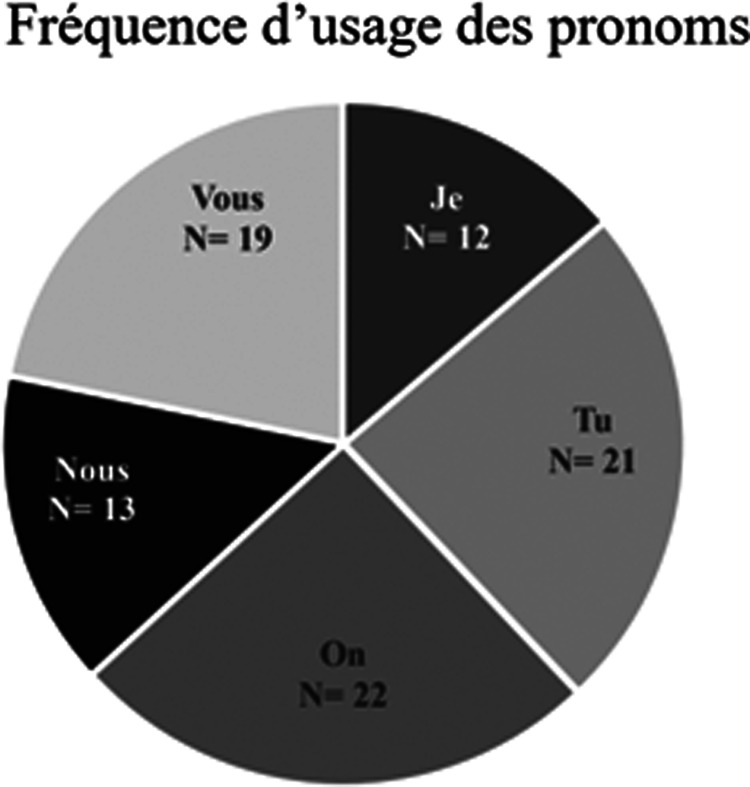
Fréquence d'usage des pronoms

L'emploi de ce dernier peut être motivé par la volonté, consciente ou inconsciente, de créer un lien entre la personne qui parle et son auditoire, et ce, de façon naturelle. Autrement dit, « l'emploi de On pour désigner une ou plusieurs personnes bien définies est surtout fréquent à l'oral dans un registre familier » (OQLF, 2020). Dans notre situation, les personnes définies sont la célébrité et ses abonné.es. Le pronom crée donc le rapprochement entre ces personnes, mais le registre de langue le fait aussi. En effet, le registre employé est celui que l'on désigne comme familier : il fait référence à la langue courante, celle parlée tous les jours avec les personnes de sa communauté. Ainsi, par l'emploi de ce registre, la célébrité fait naturellement partie du groupe ciblé puisqu'elle parle de la même façon que lui. La célébrité se montre solidaire avec son public et s'inclut dans sa situation pour lui dire qu'ils sont ensemble dans le même bateau. C'est d'ailleurs ce que nous démontre Guillaume Latendresse, un ancien joueur des Canadiens de Montréal : « On en est là, on est dans une situation qui est critique. On est à la croisée des chemins. On a encore une opportunité de sauver notre destinée. Prenons les choses en main. » (compte Twitter @*thetender1987*, 17 mars 2020). Il y a fort à parier que ce sentiment de proximité, combiné à l'influence de la célébrité, peut potentiellement avoir un effet positif sur l'acceptation du message.

Toujours par rapport aux pronoms, il a été mentionné plus tôt que les pronoms de la deuxième personne du singulier et du pluriel ont aussi souvent été utilisés. En effet, ils étaient parfois textuellement présents dans la phrase, mais ils étaient plus souvent sous-entendus dans la conjugaison de formules impératives. Par exemple, dans la chanson de Rosalie Bonenfant, elle dit : « [Ne] sous-estime pas le désinfectant pis facetime donc tes grands-parents. […] Médite si tu ne veux pas devenir folle, en autant que tu restes à la maison » (compte Facebook *Rosalie Bonenfant*, 23 mars 2020). « Sans toutefois que l'on puisse faire [du mode de l'impératif] un indicateur exact et une mesure précise de l'autoritarisme » (Petitjean, 1995, 31), il reste très intéressant de relever sa présence dans un discours public. Employer l'impératif, c'est interpeller directement son interlocuteur.trice et chercher à le.la pousser à effectuer une action que l'on préconise (Petitjean, 1995). Or, la relation entre ces deux personnes est très importante puisque « le locuteur a l'autorité que lui confère le destinataire […] » (Petitjean, 1995, 30), ce qui déterminera la suite des choses. Ainsi, pour arriver à l'objectif escompté, c'est-à-dire l'accomplissement de l'action désirée, le.la locuteur.trice doit occuper une place qui, pour différents facteurs, lui procure de l'autorité. La célébrité semble se retrouver à cet endroit en raison du capital social, de l'attention et l'influence qu'elle détient. De ce fait, les actions impératives prescrites par la célébrité risquent davantage d’être produites « dans la mesure où elle[s sont] légitime[s] » (Petitjean, 1995, 19).

Ensuite, parmi les stratégies de communication identifiées dans la grille, il y a la narration. À cet effet, on remarque plusieurs formes narratives dans les vidéos (N = 17). La narration telle que nous la percevons est l'action de raconter une histoire. Cette stratégie est singulière puisqu'elle met en scène des personnages, contrairement à la diffusion d'information où les faits sont relatés. Voici un exemple de l'application de la narration dans une des vidéos : « Je suis avec ma belle grande fille Amélia. Elle a 14 ans. Et nous, on a décidé de tourner la situation en positif et de faire l’école de la vie. Ça, c'est par exemple faire de nouvelles activités qu'on peut faire à la maison. Par exemple, on peut apprendre à cuisiner ou ça peut être de faire du ménage dans nos vieilles affaires » (compte Instagram @*mariechristine_lavoie*, 18 mars 2020). Les formes narratives présentes dans un discours ont potentiellement un effet persuasif, ce qui peut influencer le changement de comportements et d'attitudes (Harrington et al., 2015). En fait, c'est par la *transportation* (terme issu de la Transportation theory de Green & Brock, 2000, dans Harrington et al., 2015) que cela est rendu possible. La transportation est le mélange d’émotions, de ressentis, d'imagination et d'attention qui est créé et utilisé pour s'attacher à une histoire [traduction libre] (Harrington et al., 2015, 386). Elle se manifeste notamment par l'identification aux personnages qui peuvent alors devenir des modèles pour les actions de la vie réelle. Cela est d'autant plus vrai lorsque les histoires racontées sont plausibles; les écarts entre l'univers raconté et véridique semblent moins grands. Dans notre cas, lorsqu'une célébrité se met en scène et raconte une histoire, comme ses activités de confinement par exemple, il y a de fortes chances qu'une partie du public se retrouve dans son récit. Le rapprochement créé avec le personnage peut alors faire en sorte que l'on ait envie d'imiter certains comportements de celui-ci.

Pour sa part, l'autopromotion est beaucoup moins présente. En effet, il y a des formes ouvertes d'autopromotion à seulement trois reprises dans le corpus. Nous pensions que les célébrités auraient profité de cette invitation à se montrer sur les réseaux sociaux pour promouvoir leur activité ou leur produit par exemple. Comme ce n'est pratiquement pas le cas, il y a lieu de penser que ce qui compte dans cet acte de parole, c'est le message qu'il contient. La célébrité ne se fait que le.la porte-parole du message gouvernemental, et ce, peu importe l'action entreprise dans la vidéo. Encore une fois, le contexte y est certainement pour quelque chose; la crise et le sentiment de collectivité grandissant laissent peu de place aux actes individualistes. Or, si les remarques d'autopromotion ne sont pas faites ouvertement, on peut peut-être considérer la production de la vidéo comme étant une forme d'autopromotion. En adoptant cette stratégie, la célébrité se positionne positivement dans la société puisqu'elle milite pour le respect des consignes sanitaires. Il semble donc que ce soit cette vitrine qui lui permette de rehausser son image, même si à la base, il ne s'agit pas de la retombée espérée sous cette initiative.

Finalement, tel qu'il a été mentionné plus tôt, la visée dépend de l'action entreprise dans la vidéo. À cet effet, dans le corpus, les deux principales visées sont le divertissement et l'information. On remarque par ailleurs que la distribution est répartie également entre ces deux visées, comme en témoigne le graphique 6.

**Graphique 6.**
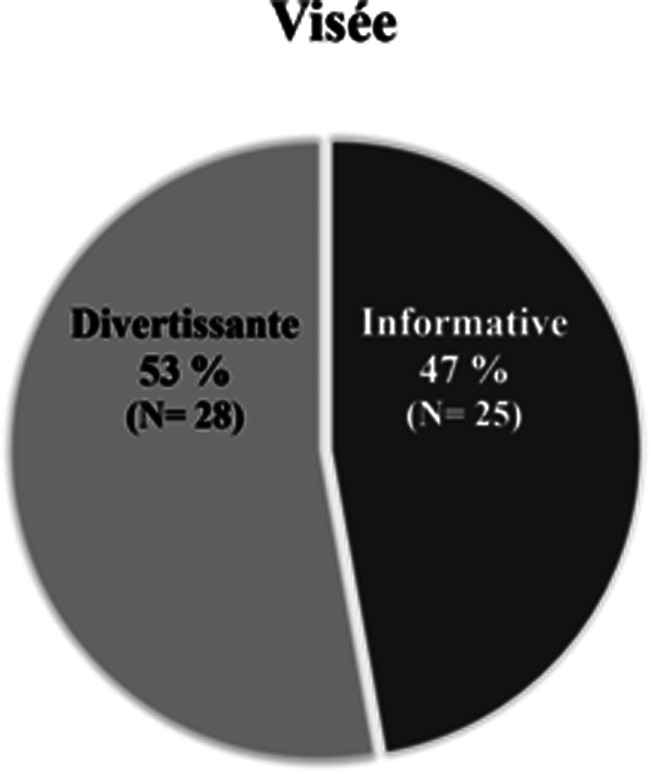
Proportion et fréquence de la visée des vidéos

Expliquons d'abord le divertissement. Lorsque quelque chose est divertissant, on dit qu'il distrait, qu'il occupe agréablement le temps (CNRTL, 2012). Ainsi, il est possible d'affirmer qu'il s'agit de divertissement lorsque la vidéo présente une chanson, une interprétation musicale, une mise en scène humoristique, un défi acrobatique ou un tour de magie. Cela étant dit, les vidéos divertissantes issues de la campagne s'inscrivent directement dans la tendance grandissante d’*infotainement*, tendance qui combine les nouvelles et l'information au divertissement (Messaris, 2019, 28). On peut penser notamment à l'intégration des consignes de santé publique à une chanson, ou au rappel des mesures sanitaires sous la forme d'une capsule humoristique pour reprendre des exemples issus de notre corpus. Cette façon de faire semble beaucoup moins moralisatrice et répressive. De plus, il faut se rappeler que la campagne a lieu sur les réseaux sociaux; ces plateformes sont utilisées pour s'informer, mais surtout pour se divertir. Ainsi, dans le but d’être vue par un maximum de personnes, la vidéo doit réussir à capter l'attention. En d'autres mots, elle doit répondre aux codes des médias socionumériques afin de devenir virale, et cela passe grandement par l’émotion positive (Eckler & Bolls, 2011). Cette charge émotive agréable, que l'on retrouve dans le divertissement, peut même mener à des changements dans les intentions et attitudes (Eckler & Bolls, 2011). Il semble donc que les artistes ont bien fait d'utiliser cette technique compte tenu du but premier de la campagne, qui est de modifier les comportements des jeunes. Finalement, il y a aussi toute la question de l'image. L'ethos d'une célébrité est respecté et préservé lorsque celle-ci produit une vidéo qui met en valeur l'objet de sa célébrité pour, en fait, parler d'autre chose. L'image doit être constante, et encore plus sur les réseaux sociaux. On remarque cette tendance plus précisément chez les chanteur.ses (qui ont bien souvent composé une chanson – voir Graphique 7) et chez les humoristes (qui ont diffusé l'information par le biais d'une mise en scène humoristique).

**Graphique 7.**
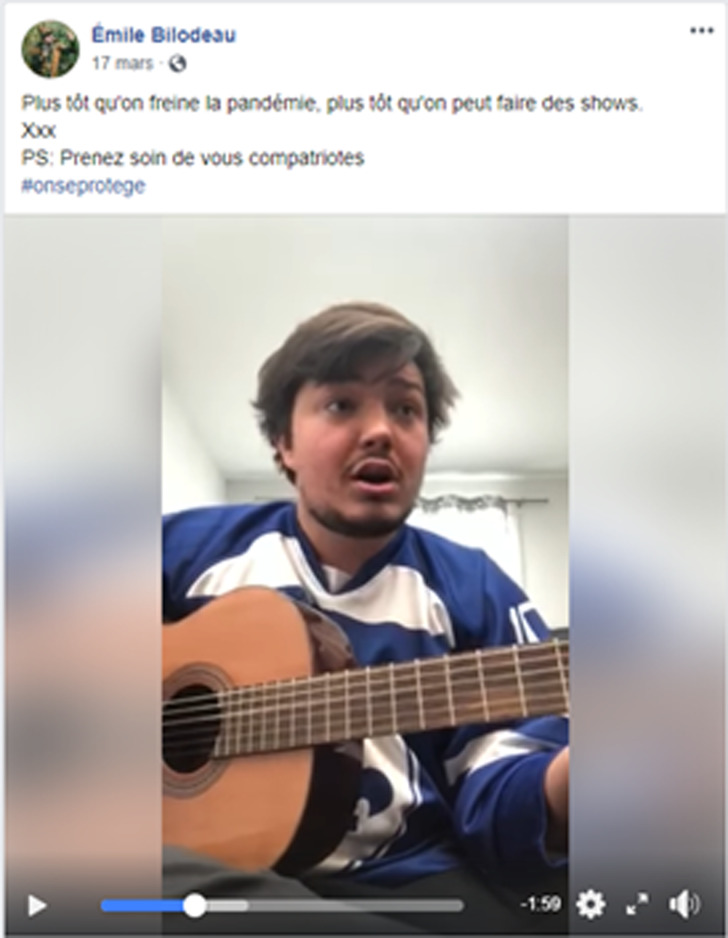
L'auteur-compositeur-interprète Émile Bilodeau joue une chanson écrite spécialement pour la situation qui s'intitule *Quarantaine*.

Pour ce qui est de la visée informative, elle se traduit dans tous les cas par une prise de parole formelle de la célébrité. En d'autres mots, il s'agit d'un discours qui a été capté par une caméra, puis diffusé sur les réseaux sociaux. Sans aucune touche d'extravagance, les célébrités qui ont opté pour ce type de vidéo se sont filmées bien souvent dans le confort de leur demeure pour s'adresser aux jeunes. Les voir ainsi, à l'intérieur de leur maison plutôt que sur les tapis rouges ou dans les galas permet d'humaniser les célébrités. Cette image les rend un peu plus « comme tout le monde », mais surtout, semblables aux jeunes qui les regardent et qui les admirent (Lalancette & Raynauld, 2019). Évidemment, cela crée un effet de proximité et de reconnaissance avec la célébrité, un lien précieux qui peut éventuellement mener à une meilleure réception du message. De plus, peut-être que la présence de la visée informative a un rapport avec la sévérité de la situation. Au moment où les vidéos ont été produites, l'heure était grave. C'est peut-être ce qui a motivé quelques influenceur.euses à produire un discours simple, clair et concis.

## Conclusion

Les résultats rapportés dans la présente étude nous en apprennent davantage sur les tendances en communication politique lors d'un appui en provenance des célébrités en contexte de pandémie. Le cas à l’étude est celui de la campagne gouvernementale #Propage l'info, pas le virus qui s'adresse aux jeunes Québécois et Québécoises. Soulignons que le cas étudié est unique en son genre. La campagne a été faite en contexte de crise et les endossements offerts par les célébrités, quelle que soit la nature de ceux-ci, se font sur une base volontaire. Autrement dit, le mouvement que les célébrités soutiennent est totalement à l'opposé de ce que l'on pourrait qualifier de *grassroots* puisqu'il est issu d'un plan gouvernemental de communication de crise. D'une certaine manière, il est possible d'avancer que ces personnalités publiques se sont senties visées et ont voulu exercer leur leadership dans un contexte où la crise sanitaire amenait à se serrer les coudes et à travailler tous ensemble pour des buts communs très louables. Certes, les personnalités publiques et influenceur.euses qui ont participé à la campagne rejoignent ce que Katz et Lazarsfeld entendent par leader d'opinion. Elles sont des personnes célèbres reconnues pour diverses raisons et du coup, elles ont tenté d'influencer leurs différents publics afin que les jeunes modifient leur comportement: ici, le respect des consignes de distanciation physique/sociale et les règles de confinement afin de ne pas propager le virus de la COVID-19. En effet, selon nos critères d'inclusion dans le cadre de cette étude, 53 célébrités ont répondu à l'appel du premier ministre Legault en produisant des vidéos et en les publiant sur leurs réseaux socionumériques afin de rejoindre les jeunes.

Il ressort des analyses que différentes façons semblent avoir accentuer le sentiment de proximité entre la célébrité et son public. L'utilisation du pronom « On », l'emploi de formules narratives et l'intimité qui se dégage des vidéos informatives vont en ce sens. Le fait que la célébrité se montre accessible et authentique peut assurer un meilleur transfert du message. Il ne faut toutefois pas oublier qu'elle détient de facto de l'influence et de l'autorité en raison de son statut; cela se reflète d'ailleurs par le mode impératif employé dans les discours. Si les célébrités ont endossé le rôle de porte-paroles du message gouvernemental, il n'en demeure pas moins qu'elles doivent quand même penser à leur image en dehors de ce contexte et, du même coup, la préserver. C'est d'ailleurs ce que les vidéos à visée divertissante rappellent. Or, même si la vidéo produite n'est pas en parfait accord avec l'ethos de la célébrité, le simple fait de participer à la campagne aura potentiellement un effet positif sur l'image, ce qui peut expliquer la faible présence d'autopromotion.

Enfin, pour revenir à la méthodologie, bien que la collecte de données à l'aide des mots-clics s'est avérée fructueuse, il y a certes une part des publications liées à la campagne et produites par les célébrités qui n'ont pas pu être recensées en raison de l'absence de l'utilisation des mots-clics. Aux yeux des influenceur.euses et des jeunes sur les médias socionumériques, peut-être que le fameux hashtag n'est plus aussi populaire qu'il a été il y a quelques années, ce qui expliquerait son absence dans les publications. Les composantes techniques des plateformes représentent aussi une limite pour la collecte de données. Facebook et Instagram proposent aux utilisateur.trices des publications sous la forme de *story* qui s'effacent après 24 heures. Il est impossible de retrouver dans les archives ces publications, à moins qu'elles aient été publiées sur un fil d'actualité régulier. Ainsi, nous pouvons supposer que nous n'avons pas eu accès à toutes les publications comme l'archivage n'a pas été fait en temps réel, ce qui a, inévitablement, des conséquences difficiles à mesurer sur le corpus de la recherche. De plus, une seule approche a été adoptée pour étudier les discours des célébrités lors de la campagne, soit l'analyse qualitative de contenu. Dans une recherche future, il serait pertinent d'analyser également les publications photographiques en lien avec la campagne qui ont été partagées sur les réseaux socionumériques afin d’étudier les imaginaires mobilisés pour convaincre les jeunes de respecter les consignes sanitaires.

Par ailleurs, il faut se rappeler que ces constats s'appliquent à un contexte sociopolitique bien précis. Le gouvernement québécois a mis à contribution de jeunes leaders pour passer un message, mais y a-t-il eu des campagnes de ce genre ailleurs dans le monde ? Est-ce que d'autres pays ont mobilisé leurs célébrités dans le contexte de la crise sanitaire et dans quel but cela a-t-il été fait ? Au mois d'avril 2020, le monde artistique international s'est regroupé pour offrir un concert « One World : Together at Home » en hommage aux travailleur.ses de la santé. Des spectacles en ligne du même genre ont aussi été produits au Canada et au Québec. Il serait plus qu'intéressant d'observer les messages politisés introduits dans ces concerts qui ont profité de cette gigantesque vitrine promotionnelle pour être diffusés.

Dans une autre ligne d'idées, la pandémie elle-même a transformé des politicien.nes et différent.es intervenant.es en célébrités en raison de leur présence constante sur les ondes, de leurs usages des médias socionumériques, mais aussi de leur personnalité. C'est notamment le cas du responsable de la santé publique du Québec, le docteur Horacio Arruda. Ses phrases chocs et sa gestuelle dynamique ont fait de lui « le héros des Québécois » (Blais, 2020). Depuis que le Québec l'a découvert, Arruda ne cesse de gagner en popularité. « Il est […] devenu une image de marque. T-shirt à son effigie, mugs et bières à son nom, gel hydroalcoolique renommé Hakuna Arruda » (Mercier, 2020). Il serait intéressant de documenter l’évolution de la célébrité de ce personnage, à savoir ce qu'il restera de ce visage principal de la crise lorsqu'elle sera terminée. Arruda arrivera-t-il à se réinventer ou à se créer une nouvelle image qui sera en mesure de rejoindre les gens autant qu'avant? Plus que jamais, on assiste à « une convergence entre les domaines de la politique, du marketing, de la culture populaire » [traduction libre] et de la communication (Marland & Lalancette, 2014, 132). Il y a lieu de poursuivre les réflexions en ce sens.

Finalement, il serait pertinent d’étudier la réception des messages auprès des jeunes. Comme il a été discuté plus tôt dans l'article, certains facteurs peuvent influencer la réception des messages des célébrités – le sujet promu, l'expertise sur le sujet, la perception de la motivation des célébrités à endosser la cause – tel que le soulignent Bergkvist et al. (2016). Il serait alors intéressant de voir comment ces facteurs ont eu un réel impact sur la réception de la campagne étudiée. Dans cette ligne d'idées et de celle des travaux de Click et al. (2015), il serait utile de se demander aussi de quelle façon le lien personnel et intime développé par les célébrités avec leurs admirateur.trices a joué un rôle sur la manière dont le message a été perçu par les jeunes (et les moins jeunes). Bref, autant de questions qui mériteraient l'attention des chercheur.es en lien avec cette pandémie qui, somme toute, a bouleversé les manières de communiquer ainsi que la vie de nombreux individus au Québec et partout dans le monde.

## Annexe I

Recension des célébrités ayant publié une vidéo sur les réseaux socionumériques.

Il s'agit des publications analysées dans le cadre de cette etude.
